# Prognostication of Learning Curve on Surgical Management of Vasculobiliary Injuries after Cholecystectomy

**DOI:** 10.1155/2016/2647130

**Published:** 2016-07-20

**Authors:** Abu Bakar Hafeez Bhatti, Faisal Saud Dar, Haseeb Zia, Muhammad Salman Rafique, Nusrat Yar Khan, Mohammad Salih, Najmul Hassan Shah

**Affiliations:** ^1^Department of HPB and Liver Transplantation, Shifa International Hospital, Islamabad 44000, Pakistan; ^2^Department of Radiology, Shifa International Hospital, Islamabad 44000, Pakistan; ^3^Department of Hepatology, Shifa International Hospital, Islamabad 44000, Pakistan

## Abstract

*Background*. Concomitant vascular injury might adversely impact outcomes after iatrogenic bile duct injury (IBDI). Whether a new HPB center should embark upon repair of complex biliary injuries with associated vascular injuries during learning curve is unknown. The objective of this study was to determine outcome of surgical management of IBDI with and without vascular injuries in a new HPB center during its learning curve.* Methods*. We retrospectively reviewed patients who underwent surgical management of IBDI at our center. A total of 39 patients were included. Patients without (Group 1) and with vascular injuries (Group 2) were compared. Outcome was defined as 90-day morbidity and mortality.* Results*. Median age was 39 (20–80) years. There were 10 (25.6%) vascular injuries. E2 injuries were associated significantly with high frequency of vascular injuries (66% versus 15.1%) (*P* = 0.01). Right hepatectomy was performed in three patients. Out of these, two had a right hepatic duct stricture and one patient had combined right arterial and portal venous injury. The number of patients who developed postoperative complications was not significantly different between the two groups (11.1% versus 23.4%) (*P* = 0.6).* Conclusion*. Learning curve is not a negative prognostic variable in the surgical management of iatrogenic vasculobiliary injuries after cholecystectomy.

## 1. Introduction

Around 750000 cholecystectomies are performed in the United States annually [1]. Laparoscopic cholecystectomy offers several advantages including less wound pain, better cosmesis, and early return to normal activity. Main disadvantage is a slightly higher risk of biliary injury than open cholecystectomy, that is, 0.5% versus 0.2% [2–4]. Variations in biliary anatomy, failure in identifying these variations, and a rising trend of performing cholecystectomy in the acute phase of inflammation may lead to more frequent occurrence of biliary injuries [1, 5]. In addition, use of laparoscopic approach not only provides environment more conducive to occurrence of iatrogenic bile duct injury (IBDI) but also increases the risk that these injuries would not be identified intraoperatively [1].

Once a biliary injury has occurred, surgical repair by experienced hepatobiliary surgeon is the most critical factor determinant of outcome [6]. It has been shown that outcomes of surgery for biliary injuries even in specialized centers have a learning curve. What constitutes a learning curve is unclear but 10–15 repairs a year have generally been referred to as “learning curve periods” by experienced centers [7, 8]. It has been shown that quality of life in patients who suffer an IBDI is compromised even after 10 years of successful intervention, costs up to 182,000 (hospital and society) pounds, and is frequently associated with malpractice litigation [9, 10]. As many as 9 different techniques have been developed to identify biliary anatomy preoperatively and intraoperatively and prevent IBDI, critical view of safety (CVS) being the one best validated [10]. With such impact of IBDI on patient lives, there are certain questions regarding associated vascular injuries in IBDI that remain unanswered. We remain unaware of the exact incidence of vascular injuries associated with biliary injuries, their impact on operative morbidity and long term biliary complications, and role of hepatectomy [11]. This raises the question that whether new HPB centers in their learning curve should embark upon IBDI associated with vascular injuries.

The objective of the current study was to demonstrate results of IBDI repair in a new HPB center during its learning phase and determine impact of concomitant vascular injuries on outcome.

## 2. Methods

We retrospectively reviewed patients who underwent surgery for iatrogenic biliary injuries at Department of HPB and Liver Transplantation, Shifa International Hospital, Islamabad, between August 2011 and December 2014. All patients were referred from other centers and no IBDI was experienced in our department. A minimum follow-up of 3 months was assured to correctly document 90-day morbidity and mortality.

All patients were seen at HPB out-patient clinic or emergency. A thorough history and physical exam were followed by relevant lab tests. We performed MRCP/ERCP for preoperative assessment of biliary tree depending upon patient's presentation and previous investigations. In addition dynamic CT scan liver was performed in all patients to assess vascular injuries and liver. These patients were discussed in a multidisciplinary team before a treatment plan was formulized. This team comprised of gastroenterologists, radiologists, and surgeons. Patients who had a failed ERCP or were not candidates for ERCP underwent surgical exploration. For classification of biliary injuries, we utilized Strasberg's classification [12]. Various biliary injuries (bile duct injuries) based on Strasberg's classification have been described as follows.

A: leak from cystic duct or an accessory duct.
B: occlusion of an accessory duct with no continuity with common bile duct.
C: leak from bile duct with no continuity with common bile duct.
D: lateral and partial injuries to main bile ducts without complete loss of continuity.
E1: complete section of common bile duct; CHD stump > 2 cm.
E2: complete section of common bile duct; CHD stump < 2 cm.
E3: no CHD available, but right and left hepatic duct confluence intact.
E4: loss of confluence with no communication between right and left hepatic ducts.
E5: aberrant right sectoral duct involved alone or in combination with CHD stricture.

For grading of complications Clavien-Dindo grading system was used [13].

We generally used right subcostal incision but, in case a patient was operated on before, scar of previous surgery was used. Roux-en-Y hepaticojejunostomy was performed in all patients and a single drain was placed near anastomosis. After operation, patients were kept in surgical step down for one day before being shifted to the ward. Broad spectrum antibiotics were administered in the postoperative period given the previous history of biliary peritonitis or obstructive jaundice.

For the purpose of this study, patients were divided into two groups, that is, Group 1 IBDI and Group 2 IBDI with vascular injury. The two groups were compared for variables including demographics, predominant symptoms, past history of surgeries, and endoscopic intervention. Operative variables including type of biliary injury, associated vascular injuries, and type of repair were also compared. Outcome was assessed on basis of 90-day morbidity and mortality. Categorical variables were assessed using chi square and Fischer's test while *t*-test was used for interval variables. SPPS version 20 was used for statistical analysis. The study was performed in accordance with declaration of Helsinki. It was a noninterventional study and no potential identifiers were present. Hospital ethics committee granted exemption from formal review of this study (IRB number 582-030-2016).

## 3. Results

A total of 39 patients underwent surgical management of IBDI. Median age of our cohort was 39 (20–80) years. Male-to-female ratio was 1 : 5.5. Median time to cholecystectomy and presentation was 72 (3–920) days in patients with associated vascular injury and 312 (5–5436) days in patients without vascular injury and was not significantly different (*P* = 0.5). There were 9 (23%) patients with concomitant vascular injuries. No difference was observed between Groups 1 and 2 with respect to gender, presenting symptom, surgical access, and radiological interventions as shown in Table 1.

### 3.1. Operative Details

Table 2 demonstrates types of vascular and biliary injuries in our patients. Based on Strasberg's classification of biliary injuries, 27 (69.2%) patients had E3 and E4 injuries. All patients underwent Roux-en-Y hepaticojejunostomy. There were 9 (23%) patients with 10 (25.6%) vascular injuries. All patients except 1 had injury to right vascular structures. In this patient left portal vein was also injured and thrombosed along with right hepatic artery. She was managed with hepaticojejunostomy and PTFE graft from main portal vein to left portal vein. Only one patient underwent right hepatectomy due to combined arterial and portal venous injury. Other patients with vascular injuries were managed with HJ alone. A right hepatectomy was performed in three patients. Out of these, two had a right hepatic duct stricture associated with right lobe atrophy and one patient had combined right arterial and portal venous injury with resultant liver infarction.

### 3.2. Outcome

Mean follow up time was 8 ± 8.3 months and ranged between 3 to 34 months. Mean hospital stay was 6.1 ± 2.1 days. Overall 90 day morbidity was 10 (25.7%) and there were only 3 (7.6%) grade III and above complications as shown in Table 3. There was no mortality.

### 3.3. Comparison of Surgical Details

Out of 39 patients, who suffered an IBDI, 11 (28.2%) had previous history of laparoscopic cholecystectomy. In only 4 (10.2%) patients, a biliary injury was recognized intraoperatively. Around half of all patients (51.3%) were explored at least once for surgical repair of biliary injury before they were referred to us. Out of these 11 patients underwent a hepaticojejunostomy, that is, 9 at the first surgery and 2 in the second exploration.  Table 4 represents various surgical variables compared between the two groups. E2 injuries were associated with a high frequency of vascular injuries (66% versus 15.1%) (*P* = 0.01). The number of patients who developed postoperative complications was not significantly different between the two groups. All patients who underwent hepatectomy had an underlying vascular injury (*P* = 0.009).

## 4. Discussion

Vascular injuries are frequently associated with biliary injuries after cholecystectomy. Although majority of them can be managed expectantly, some require major surgical intervention. Roux-en-Y hepaticojejunostomy represents an excellent surgical technique even in patients with previous failed attempts at bile duct repair. Limitations of the current study include its retrospective design, potentially missed preoperative and postoperative variables, and relatively short follow-up. In addition a multivariate analysis of independent prognostic factors could not be performed due to low numbers of observed complications.

We classified biliary injuries based on Strasberg's classification which is a well renowned classification system [12]. A number of other classifications exist in the literature [14–16]. Complex anatomy of portal region, frequent variations in anatomy, multitude of injury mechanisms, and diagnostic and treatment modalities available have produced a spectrum of biliary injuries that cannot be fully explained by any single classification.

Certain variations in biliary anatomy predispose to iatrogenic injuries after cholecystectomy. Anatomical variations in biliary anatomy might be seen in as high as 20% patients undergoing cholecystectomy [17, 18]. Cystic artery and duct anomalies are the most frequent and might be seen in up to 15% patients. Cystic duct opens at variable levels on bile duct. A short cystic duct makes its identification difficult and also predisposes to clip slippage whereas a long cystic duct might be confused with CBD. A short cystic artery risks damage to right hepatic artery. Among variations in hepatic arterial anatomy, Moynihan's hump is one of the most significant and predisposes to uncontrollable bleeding, misidentification with clipping, and stricture formation. Development of CVS is very important to minimize risk of IBDI. This involves dissection of Calot's triangle from all fatty tissue, mobilization of lowest part of gall bladder, and unambiguous identification of cystic duct and artery entering gall bladder [10].

The true incidence of concomitant vascular injuries with IBDI is not well known but can range between 12 and 61% [3, 19, 20]. This variation primarily is representative of differences in patient groups included in these studies. There were 9 (23%) patients with 10 (25.6%) vascular injuries in the current study. Although majority of patients were managed expectantly in terms of their vascular injury, two patients required an additional surgical procedure including hepatectomy and PTFE graft reconstruction. We advocate routine use of dynamic liver CT to properly identify vascular injuries and assess liver status before a surgical attempt is finalized. We found a statistically significant association between vascular injury and E2 injuries. It has been shown that majority of vascular injuries that occur alongside biliary injury are E1/E2. That is because the RHA usually skirts around the common hepatic duct at this level. However, at the time of final intervention many injuries have progressed to E3/E4 levels depending upon exact level of biliary ischemia [21, 22]. That probably is why E3/E4 injuries were more frequent in the current study. Impact of vascular injury on outcome is also a matter of debate with studies reporting conflicting results [23–29]. With this ambiguity it is difficult for a new HPB center in its learning curve to ascertain whether to embark upon bile duct repairs with vascular injuries or refer these cases to more experienced centers. In the current study, there was no difference in postoperative complication rate between patients with and without concomitant vascular injuries. A postop complication rate between 20 and 26% and hospital mortality of 3% have been shown with IBDI repairs [7, 8, 30–32]. We had a comparable complication rate, no anastomotic leak/stricture was observed, and the hospital mortality was zero. Several factors might have played a pivotal role in achieving these acceptable results. It has been shown that skills acquired in living donor liver transplant setting could facilitate and ease out complex biliary surgeries [33, 34]. It is possible that, as >20 transplants/year, we had achieved effective technical skills in dealing with biliary injuries due to our living donor liver transplant experience. Use of fine sutures like 7/0 and 8/0 prolene and PDS, preservation of microcirculation of bile duct, and making anastomosis under loupe magnification in LDLT allow better understanding of portal anatomy and refinement in surgical technique. A multidisciplinary approach with thorough discussion with interventional gastroenterologists and radiologists allowed better understanding of extent of biliary injury, exact level of injury, and whether vascular structures were involved or not. A dynamic CT scan accurately identified liver status, integrity of hepatic arteries and portal vein, and possibilities of reconstruction in the event where there was a vascular injury. It also helped us in identifying patients who would need a liver resection. Surgeon's experience was a crucial factor as the primary surgeon had more than 10-year experience in dealing with various types of hepatobiliary cases and construction of biliary anastomosis. Follow-up in the current study is relatively short and our results do not reflect upon long term outcomes. An element of follow-up loss in our patients cannot be excluded as they were referred from remote regions of the country and once they resumed their normal life, they did not seek follow-up. Strasberg et al. suggested that patients with an underlying vascular injury who undergo biliary repair within days are more likely to develop anastomotic strictures than patients who are operated on later [11, 35]. Out of 9 patients in Group 2, two had a repair at day 3 and day 4 while the rest were operated on at least after 4 or more weeks.

Combined vasculobiliary injury may lead to slow atrophy of right lobe of liver [36, 37]. Atrophy is more likely to occur with E4 injuries since they disrupt hilar collaterals from the left hepatic artery in case of right hepatic arterial injury [9, 37]. Three patients required right hepatectomy in our series. All had E4 injuries. Underlying arterial injury was present in all of them while one patient had a combined hepatic arterial and portal venous injury.

## 5. Conclusion

The current study demonstrates acceptable surgical outcomes from a new HPB center in management of complex biliary injuries. Concomitant vascular injuries can be effectively managed during learning curve and an active liver transplant program may help in achieving improved outcomes. Dynamic CT scan should be performed in all patients to correctly assess vascular status. A multidisciplinary approach should be taken and long term follow-up of patients with vasculobiliary injury should be performed to identify late complications and assess quality of life.

## Figures and Tables

**Table 1:** Patient characteristics.

		IBDI with vascular injury		IBDI		*P* value
		*N* = 9		*N* = 30		
		Number	Percent	Number	Percent	
Gender	Male	1	11.1	5	16.6	1.0
	Female	8	88.9	25	83.4	

Presenting symptom	Abdominal pain	5	55.5	14	46.6	0.4
	Bile in drain	1	11.1	1	3.4	
	Jaundice	3	33.4	15	50	

Cholecystectomy	Laparoscopic	2	22.2	9	30	1.0
	Open	7	77.8	21	70	

Radiological intervention	ERCP	6	66.7	23	76.6	0.2
	PTC	1	11.1	0	0	

**Table 2:** Biliary and vascular injuries.

		Number	Percent
Type of injury	C	2	5.2
	D	3	7.7
	E1	1	2.5
	E2	6	15.4
	E3	20	51.3
	E4	7	17.9

Type of vascular injury	Right PV ligated	1	2.6
	Left portal vein thrombosis	1	2.6
	Right PV thrombosis	1	2.6
	Right hepatic artery clipped	3	7.8
	Right hepatic artery ligated	4	10.2

**Table 3:** Outcome based on 90-day morbidity.

			Number	Percent
Morbidity	Grade II	Wound infection	7	17.9
	Grade III A	Pleural effusion	2	5.2
	Grade IV	Sepsis	1	2.6

**Table 4:** Comparison between patients IBDI with and without vascular injury.

		IBDI with vascular injury		IBDI		*P* value
		*N* = 9		*N* = 30		
		Number	Percent	Number	Percent	
Previous surgical attempts	Yes	3	33.4	18	60	0.2
	No	6	66.6	12	40	

IBDI recognized intraoperatively	Yes	1	11.1	4	13.4	1.0
	No	8	88.9	26	86.6	

Type of previous surgery	Drain placement	1	11.1	10	33.3	0.5
	Hepaticojejunostomy	2	22.2	7	23.4	

Number of surgical attempts	1	2	22.2	8	26.8	0.5
	2	1	11.1	10	33.3	

Vasculobiliary injury association	E2	4	44.5	2	6.7	0.01
	Others	5	55.5	28	93.3	

Final surgery	Hepaticojejunostomy	7	77.7	21	70	0.6
	Redo HJ	2	22.3	9	30	

Hepatectomy	Yes	3	33.3	0	0	0.009
	No	6	66.7	30	100	

Complications	No	8	88.9	23	76.6	0.6
	Yes	1	11.1	7	23.4	
